# Variations in Spike Glycoprotein Gene of MERS-CoV, South Korea, 2015

**DOI:** 10.3201/eid2201.151055

**Published:** 2016-01

**Authors:** Dae-Won Kim, You-Jin Kim, Sung Han Park, Mi-Ran Yun, Jeong-Sun Yang, Hae Ji Kang, Young Woo Han, Han Saem Lee, Heui Man Kim, Hak Kim, A-Reum Kim, Deok Rim Heo, Su Jin Kim, Jun Ho Jeon, Deokbum Park, Joo Ae Kim, Hyang-Min Cheong, Jeong-Gu Nam, Kisoon Kim, Sung Soon Kim

**Affiliations:** Korea Centers for Disease Control and Prevention, Cheongju-si, South Korea

**Keywords:** Middle East respiratory syndrome coronavirus, MERS-CoV, viruses, Middle East respiratory syndrome, spike glycoprotein gene, genetic evolution, recombination, outbreak, South Korea

## Abstract

An outbreak of nosocomial infections with Middle East respiratory syndrome coronavirus occurred in South Korea in May 2015. Spike glycoprotein genes of virus strains from South Korea were closely related to those of strains from Riyadh, Saudi Arabia. However, virus strains from South Korea showed strain-specific variations.

Since it was first identified in 2012, Middle East respiratory syndrome coronavirus (MERS-CoV) has emerged as a novel viral pathogen that causes severe acute respiratory illness, including fever, cough, and shortness of breath (*1*). The current outbreak of infection with this virus in South Korea, which began on May 20, 2015, has infected 186 patients and caused 36 deaths within 2 months. This developing public health concern has attracted worldwide attention as a potential cause of a global pandemic. Although extensive biologic and clinical characterization should be performed to measure the public health effect of this outbreak, currently available genetic data are informative in clarifying virus alterations that affect transmissibility.

MERS-CoV spike (S) glycoprotein binds cellular receptor dipeptidyl peptidase 4 (DPP4, CD26) for host cell entry (*2*), and a receptor-binding domain (RBD) on virus S protein mediates this interaction (*3*). In addition, S proteins expressed on the virus surface can induce host antibodies that block MERS-CoV entry (*4*). To investigate changes in the S gene associated with viral evolution and possible genetic markers of altered transmissibility, an analysis of S genes obtained from clinical specimens during the early phase of the outbreak was performed.

## The Study

We identified genetic variability of MERS-CoV S genes among infected persons in South Korea. Samples from 7 patients identified as positive for MERS-CoV were sequenced. These patients were identified by using sequences upstream of the envelope protein gene and open reading frame (ORF) 1a in real-time reverse transcription PCRs (*5*) (Table 1).

**Table 1:** Sequence information for Middle East respiratory syndrome coronaviruses isolated from 8 patients, South Korea, May 2015*

Patient	Sequence	Date of symptom onset/sample collection	Sample	Sequencing method	GenBank accession no.
PAT001	CoV/KOR/KNIH/001_05_2015	11/19	Sputum	Illumina,† Sanger	KT182958
PAT002	CoV/KOR/KNIH/002_05_2015	19/20	Third-passage isolate from Vero cells	Illumina	KT029139
PAT009	CoV/KOR/KNIH/009_05_2015	27/28	Sputum	Illumina	KT182953
PAT010	ChinaGD01‡	19/27	Nasopharyngeal swab	Ion torrent,§ Sanger	KT006149
PAT012	CoV/KOR/KNIH/012_05_2015	21/28	Sputum	Sanger	KT182954
PAT013	CoV/KOR/KNIH/013_05_2015	21/28	Sputum	Sanger	KT182955
PAT015	CoV/KOR/KNIH/015_05_2015	22/30	Sputum	Illumina	KT182956
PAT042	CoV/KOR/KNIH/042_05_2015	25/30	Sputum	Illumina	KT182957

*MERS-CoV, Middle East respiratory syndrome coronavirus. †Illumina (San Diego, CA, USA). ‡Sequence obtained from the Chinese Centers for Disease Control and Prevention (Beijing, China). §Life Technologies (Bleiswijk, the Netherlands).

**Figure 1:**
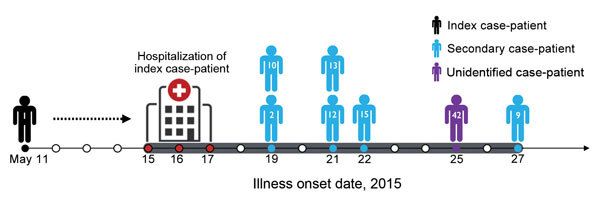
History of confirmed cases of Middle East respiratory syndrome coronavirus infection, South Korea, May 2015. Eight confirmed cases of human infection with this virus are shown according to date of onset of illness. The unidentified case-patient was a patient for whom the transmission source was not identified. Red circles in time line indicate hospitalization period for the index case-patient. Black, blue, and purple circles in time line indicate recorded symptom onset date for each patient. Numbers within human symbols are patient numbers.

Index case-patient 1 (PAT001) had traveled to Bahrain, the United Arab Emirates, and Saudi Arabia during April 24–May 4, 2015, and became symptomatic on May 11 after his return to South Korea (*6*). After he visited a local clinic, his symptoms worsened, and he was hospitalized on May 15. During his hospitalization (May 15–17), PAT001 shared a room with PAT003 and the same ward with PAT009, PAT012, PAT013, and PAT015. PAT042 was admitted to the same hospital on May 19 (Figure 1). PAT010 was the son of PAT003 and had visited his father in the hospital before traveling to China, where he became symptomatic and tested positive for MERS-CoV. PAT002 was the wife of PAT001 and cared for him during his illness.

The S gene was amplified directly from nucleic acids extracted from respiratory specimens (6 patients) or a viral isolate (1 patient) (*7*) by using the QIAamp Viral RNA Mini Kit (QIAGEN, Hilden, Germany). Reverse transcription was performed by using the Superscript III First-Strand Synthesis System (Life Technologies, Bleiswijk, the Netherlands) and virus-specific reverse primers. cDNA was amplified by using an overlapping PCR to generate products of 600–3,000 bp that covered the entire S gene.

Resulting PCR amplicons were sequenced by using Sanger sequencing with an ABI 3730 Analyzer (Applied Biosystems, Foster City, CA, USA) or next-generation sequencing. For next-generation sequencing, PCR products were pooled and fragmented to an average of 300 bp, and a sequencing library was constructed by using the Illumina TruSeq Nano DNA Sample Prep Kit (Illumina, San Diego, CA, USA). Sequencing was performed by using the Illumina MiSeq Platform (Illumina).

To explore relationships of newly isolated virus strains from South Korea with other MERS-CoV strains, 131 reference MERS-CoV S gene sequences from GenBank and MERS-CoV Sequences June 2015 (http://tinyurl.com/MERS-CoV-4Jun15) (*8*) and 8 strains from South Korea, including a sequence from PAT010 (ChinaGD01; Chinese Centers for Disease Control and Prevention, Beijing, China), were aligned by using MUSCLE software (*9*). This alignment was used for subsequent phylogenetic analysis. A phylogenetic tree was constructed by using the maximum-likelihood method with a bootstrap value of 1,000 and RAxML software (*10*).

**Figure 2:**
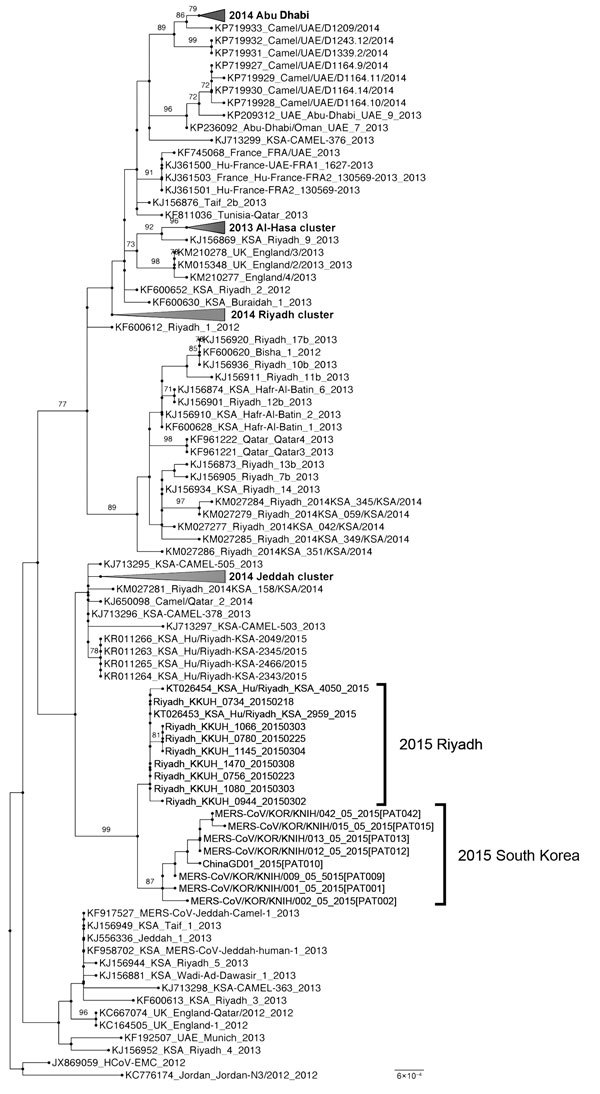
Molecular phylogenetic tree and coding region variants for spike glycoprotein genes of Middle East respiratory syndrome coronavirus (MERS-CoV) isolates from South Korea, May 2015, and reference MERS-CoV sequences. Phylogenetic analysis of 139 spike glycoprotein gene sequences was performed by using RAxML software (*10*). Tree was visualized with FigTree v.1.4 (http://tree.bio.ed.ac.uk/software/figtree). Taxonomic positions of circulating strains from the outbreak in South Korea and Riyadh are indicated. Compressed major clades of MERS-CoV are indicated in bold. Bootstrap values (>70%) on nodes are shown as percentages on the basis of 1,000 replicates. Scale bar indicates nucleotide substitutions per site.

All 8 ORFs from virus isolates obtained during the outbreak in South Korea were most closely related to ORFs of the recently isolated 2015 Riyadh clade, but isolates from South Korea constituted a novel branch, which was supported by a bootstrap value of 87% (Figure 2). Phylogenetic data indicated that virus isolates from other patients originated from virus isolates from the index case-patient. These data also showed that strains detected in 2015 formed 2 groups: KSA-2466–like viruses and KKUH_0734–like viruses. Viruses from South Korea isolated in 2015 clustered with 1 sublineage of KKUH_0734–like viruses from Saudi Arabia.

Nucleotide sequence comparisons with 131 reference MERS-CoV S genes showed that the clade from South Korea had highest identity (99.68%–99.9%) with recently circulating strains from Riyadh isolated in 2015. Strains from South Korea had 8 novel nucleotide substitutions (C183G, A409C, T1586C, G1588C, T1848C, G1886A, T3177C, and C3267T) that are unique to the South Korea lineage and share nucleotide substitution T258C with some viruses from Saudi Arabia detected earlier in 2015 (Table 2). T3177C and C3267T mutations were observed only in all viruses from South Korea.

**Table 2:** Genetic changes in spike glycoprotein gene sequences strain-specific variants of MERS-CoV from South Korea compared with those of other MERS-CoV isolates*

Virus isolate	Nucleotide (amino acid) positions								
	NTD			RBD		Other regions			
183	258	409	1586	1588	1848	1886	3177	3267
(61)	(86)	(137)	(529)	(530)	(616)	(629)	(1059)	(1089)
JX869059_HCoV-EMC_2012	C (G)	T (V)	A (S)	T (I)	G (V)	T (V)	G (R)	T (D)	C (S)
KR011266_KSA_Hu/Riyadh-KSA-2049/2015	•	•	•	•	•	•	•	•	•
KR011263_KSA_Hu/Riyadh-KSA-2345/2015	•	•	•	•	•	•	•	•	•
KR011264_KSA_Hu/Riyadh-KSA-2343/2015	•	•	•	•	•	•	•	•	•
KR011265_KSA_Hu/Riyadh-KSA-2466/2015	•	•	•	•	•	•	•	•	•
KT026453_KSA_Hu/Riyadh_KSA-2959_2015	•	C (V)	•	•	•	•	•	•	•
Riyadh_KKUH_0734_20150218	•	C (V)	•	•	•	•	•	•	•
Riyadh_KKUH_0756_20150223	•	C (V)	•	•	•	•		•	•
Riyadh_KKUH_0780_20150225	•	C (V)	•	•	•	•	•	•	•
KT026454_KSA_Hu/Riyadh_KSA_4050_2015	•	C (V)	•	•	•	•	•	•	•
Riyadh_KKUH_0944_20150302	•	C (V)	•	•	•	•	•	•	•
Riyadh_KKUH_1066_20150303	•	C (V)	•	•	•	•	•	•	•
Riyadh_KKUH_1080_20150303	•	C (V)	•	•	•	•	•	•	•
Riyadh_KKUH_1145_20150304	•	C (V)	•	•	•	•	•	•	•
Riyadh_KKUH_1470_20150308	•	C (V)	•	•	•	•	•	•	•
MERS-CoV/KOR/KNIH/001_05_2015 [PAT001]	•	C (V)	•	C (T)	•	•	•	C (D)	C (S)
MERS-CoV/KOR/KNIH/009_05_2015 [PAT009]	•	C (V)	•	•	•	C (V)	•	C (D)	C (S)
ChinaGD01_2015 [PAT010]†	G (C)	C (V)	•	•	•	C (V)	•	C (D)	C (S)
MERS-CoV/KOR/KNIH/012_05_2015 [PAT012]	•	C (V)	•	C (T)	•	C (V)	•	C (D)	C (S)
MERS-CoV/KOR/KNIH/013_05_2015 [PAT013]	•	C (V)	•	C (T)	•	C (V)	•	C (D)	C (S)
MERS-CoV/KOR/KNIH/015_05_2015 [PAT015]	•	C (V)	•	C (T)	•	C (V)	•	C (D)	C (S)
MERS-CoV/KOR/KNIH/042_05_2015 [PAT042]	•	C (V)	•	C (T)	•	C (V)	A (H)	C (D)	T (S)
MERS-CoV/KOR/KNIH/002_05_2015 [PAT002]‡	•	C (V)	C (R)	•	C (L)	•	•	C (D)	T (S)

*Alphanumeric codes in brackets indicate patient (PAT) number. Dots indicate no change in sequence identity. MERS-CoV, Middle East respiratory syndrome coronavirus; NTD, N-terminal domain; RBD, receptor-binding domain. Dots indicate sequence identity. †MERS-CoV strain ChinaGD01 (KT006149). ‡Third-passage isolate from Vero cells.

Of the 8 nucleotide substitutions, 4 (A409C, T1586C, G1588C, and G1886A) were nonsynonymous and resulted in 4 amino acid changes (S137R, I529T, V530L, and R629H) (Table 2). Among these mutations, 2 nonsynonymous variants (S137R and V530L) were identified in isolates from PAT002 after the third passage in Vero cells and were assumed to be cell culture–adaptive mutations (*11*). The I529T and V530L mutations were located in the RBD, but not at the RBD–DPP4 receptor interface (*3*). The R629H mutation was situated outside the RBD. However, on the basis of only these results, we could not determine whether these amino acid substitutions affected receptor-binding affinity between human DPP4 receptor and MERS-CoV S protein.

To understand the rate at which virus genetic diversification occurred during the outbreak in South Korea, we used the Bayesian–Markov Chain Monte Carlo method in BEAST version 2.1.3 (http://beast2.org/) for 8 S genes. The Hasegawa, Kishino, and Yano substitution model was selected under uncorrelated lognormal molecular clock and a birth–death coalescent.

The S gene was estimated to evolve at mean rate of 6.72 × 10^−3^ substitutions/site/year (95% highest posterior density [HPD] 5.59−6.93 × 10^−3^ substitutions/site/year). This mutation rate for the S gene was higher than that for complete MERV-CoV genomes in other studies: 1.12 × 10^−3^ substitutions/site/year (95% HPD 8.76 × 10^−4^−1.37 × 10^−3^ substitutions/site/year) (*12*) and 9.29 × 10^−4^ substitutions/site/year (95% HPD 7.19 × 10^−4^−1.15 × 10^−3^ substitutions/site/year) (*13*). However, more data are required to demonstrate the pattern of MERS-CoV evolution during the outbreak in South Korea because results are limited by a relatively low number of sequences, short selected time points, examination of only the S gene region, and different sequencing methods.

## Conclusions

Accurate genome sequencing can identify spatiotemporal patterns that help understand dynamics of rapid spread of MERS-CoV infection. We report S glycoprotein gene sequences of MERS-CoV from 8 patients and a strain cultured in Vero cells. Genetic information obtained is useful for understanding the evolutionary history of MERS-CoV.

On the basis of our phylogenetic analyses, virus sequences of strains isolated in South Korea in 2015 form a unique clade. Genetic variations elucidated in this study show an unreported sequence in the RBD, which suggests that MERS-CoV circulating in South Korea during the outbreak in 2015 has higher genetic variability and mutation rates. However, we cannot conclude that deleterious effects promoting spread of infection will occur because of these mutations. Additional genetic information will resolve precise characteristics of the MERS-CoV obtained during the outbreak in South Korea.
